# The Emergence of Personalized Health Technology

**DOI:** 10.2196/jmir.5357

**Published:** 2016-05-10

**Authors:** Luke Nelson Allen, Gillian Pepall Christie

**Affiliations:** ^1^ Harvard T.H. Chan School of Public Health Department of Global Health and Population Boston, MA United States; ^2^ Vitality Group New York, NY United States

**Keywords:** personalized health technology, population health, frugal innovation, ethics, socioeconomic factors, inequalities, technology, health

## Abstract

Personalized health technology is a noisy new entrant to the health space, yet to make a significant impact on population health but seemingly teeming with potential. Devices including wearable fitness trackers and healthy-living apps are designed to help users quantify and improve their health behaviors. Although the ethical issues surrounding data privacy have received much attention, little is being said about the impact on socioeconomic health inequalities. Populations who stand to benefit the most from these technologies are unable to afford, access, or use them. This paper outlines the negative impact that these technologies will have on inequalities unless their user base can be radically extended to include vulnerable populations. Frugal innovation and public–private partnership are discussed as the major means for reaching this end.

## Introduction

Several larger technology giants and smaller upstarts are creating personalized health technologies. Sensors, smartwatches, and mobile health apps are strapped to wrists and placed in pockets to monitor and help to modify health behaviors [1]. High-profile devices include the Fitbit, Jawbone, Microsoft Band, and Apple smartwatch. While there is no widely agreed-upon definition, personalized health technology generally refers to wearable devices that monitor health-related activity and provide feedback at the individual level, usually through a corresponding app or minidisplay on the device. Current products track lifestyle information such as steps walked, hours slept, and calories consumed. Terabytes of data are analyzed to deliver instantaneous and predictive insights to users. These technologies contribute to the self-quantification movement and to the consumerization of health.

PHTs that empower consumers to quantify health behaviors could advance health for all populations. The modifiable risk factors that are measured by these devices—including physical activity and diet—are major drivers of noncommunicable diseases. These conditions, including cardiovascular and lung diseases, type 2 diabetes, and various cancers, are the leading causes of death and disability worldwide [2]. They account for an estimated 60% of all deaths and will cost the global economy US $30 trillion by 2025 [2,3].

Scientific evidence demonstrating the impact of personalized health technologies on health is still emerging. This is partly because rapid technological innovation is being driven by firms responding to a market for these products, leaving academics and clinicians to play catch-up with health impact evaluations. Early research suggests that the technologies can facilitate changes in behaviors and reductions in disease risks, and that the health impact is magnified when coupled with broader engagement strategies [4-6]. Although noncommunicable diseases disproportionately afflict disadvantaged groups [2,7,8], the uptake of personalized health technologies has been limited to the educated, healthy, and wealthy [9-11]. Devices remain largely unaffordable and inaccessible to lower-income populations, and many lack the technological skills required to modify their health behaviors using information delivered by the technologies [12-14]. Until access is expanded to nontraditional users, personalized health technologies will continue to widen socioeconomic health inequities for vulnerable populations worldwide.

## The Problem: Exacerbating Inequity

Inequities are often exacerbated in the shorter term when innovative technologies enter the marketplace. Companies place higher prices on new technologies to recover their original investment in research and development. Over time, the power of economies of scale and competitive forces push prices downward: technologies eventually become affordable to the masses. As relatively new products, personalized health technologies have served to widen inequities because only affluent early adopters can afford their higher prices, while marginalized populations remain excluded [10,15].

Personalized health technologies further exacerbate inequities in the shorter term because early adopters are motivated and health conscious [11]. Existing users tend to be highly educated and possess the necessary technological skills to operate the devices. They also have the linguistic and numeric capabilities to process information in order to change behaviors. While high-income earners possess these skills, vulnerable populations—older adults, racial and ethnic minorities, poorly educated individuals, and low-income earners—commonly lack them [16]. Without adequate technological or health literacy skills, marginalized populations cannot actively engage with personalized health technologies. These factors may widen socioeconomic health inequities further in the short term [15].

How can gaps in the affordability and accessibility of personalized health technologies be closed in the longer term? According to Tudor Hart’s inverse care law, health products and services are always used most by those who need them least [17]. As devices become more efficacious, advancements in health will continue to disproportionately benefit the privileged. Personalized health technology will not realize its public health potential in reducing the global burden of noncommunicable diseases unless challenges associated with the affordability and accessibility of personalized health technologies are proactively mitigated.

## The Solution: Frugal Innovation

Creative strategies are required to advance health for individuals occupying lower rungs of the socioeconomic ladder. Innovating for the bottom of the pyramid—the 3 billion people living on less than US $2.50 a day—is not new [18]. In 2002, the renowned management professor CK Prahalad proposed the development of products and services for the bottom of the pyramid. Prahalad realized that fortunes were being left on the table as companies neglected to target the largest but poorest socioeconomic population [19]. Since Prahalad’s writings, innovating with a frugal innovation mindset has emerged to target these previously marginalized consumers.

Frugal innovations are high-quality products created with limited resources [20]. Innovating with a frugal innovation mindset entails reducing the cost and complexity of products by removing nonessential features to create “good enough” products. Materials are repeatedly recycled to self-sustain the company and the environment, while diverse external partners such as universities and venture capitalists are often brought together to maximize efficiencies. The needs and requirements of end users in bottom-of-the-pyramid markets are central to the development process.

This relatively new model has received widespread support from socially oriented enterprises and influential corporate leaders alike. Unilever’s Chief Executive Officer, Paul Polman, wrote in the foreword of Radjou and colleague’s book *Frugal Innovation* that the “frugal ingenuity of developing nations with the advanced [research and development] capabilities of advanced economies [can enable] companies to create high-quality products and services that are affordable, sustainable, and benefit humanity as a whole” [20]. Indra Nooyi, Chairperson and Chief Executive Officer of PepsiCo, further contends that “frugal innovation is one of the most critical emerging models of value creation for both businesses and the customers they serve” [20].

Numerous companies have created products and services for health using this approach [21,22]. General Electric has developed an electrocardiograph machine that costs US $800 as opposed to US $2000 and has reduced the cost of an electrocardiographic test to US $1 per person. Tata has established the Tata Swach to purify water without running water or electricity for US $20. The innovative Jaipur foot is a prosthetic that costs less than US $45 [23]. A majority of these innovations are widely used in developing as well as in developed countries. They also enable developing countries to leapfrog their developed country counterparts to provide cost-effective innovations at scale.

Despite the emergence of frugal innovations, challenges arise that could hinder their broader uptake and use. Predicting what consumers need and desire is relatively easier than actually engaging them, particularly when the target group is a marginalized population. In addition, balancing financial and social returns to sell at scale while continuing to generate profits is a ubiquitous issue for companies engaged in frugal innovation.

It is also important that emerging systems, tools, and personalized health technology devices be subject to rigorous technology assessments [24]. Established qualitative and quantitative tools [25] can be used to evaluate performance against a range of key performance indicators that extend beyond health metrics to include reliability, integration with other devices, cost, and data security [24].

## Frugal Innovation and Personalized Health Technology

A frugal innovation mindset can be applied to personalized health technologies to minimize socioeconomic health inequities. Companies can engage end users in bottom-of-the-pyramid markets to design, develop, and test the effectiveness of personalized health technologies. There are several ongoing pilot projects that use personalized health technologies to improve health outcomes in low-income populations [26-28]. Unfortunately, the majority of early personalized health technology initiatives in low- and middle-income countries tended to lack careful targeting, sustainable funding, robust evaluation, and the ability to work at scale [29-31]. In addition to these projects, more affordable personalized health technologies and programs are emerging to benefit bottom-of-the-pyramid markets, including the Xiaomi Mi Band and United Nations Children’s Fund (UNICEF) Kid Power Bands. In a recent study, the Mi Band—sold in Asia and priced at less than US $20—outperformed more expensive competitors in accurately monitoring vital signs and steps walked [32]. Another innovative example of frugal personalized health technology that benefits low-income groups in both high- and low-income countries is the UNICEF Kid Power Program. UNICEF and the US retailer Target market the discounted Kid Power Band (a wrist-worn activity-monitoring device) at children in high-income countries with obesity problems. By meeting physical activity goals, wearers earn points, recorded on a parallel mobile app. Corporate sponsors translate these points into funding for childhood nutrition programs in developing countries. To date, over 50,000 children have engaged with the initiative, raising enough money to provide over a quarter of a million UNICEF therapeutic food packets for malnourished children [33].

As evidence for the effectiveness of these technologies mounts, governments with legal and constitutional obligations to promote the health of their citizens can subsidize the creation of personalized health technologies and provide them through state-funded programs. These measures will help to mitigate the inequities that this disruptive technology is exacerbating.

## Conclusion: A Call for Collective Action

Technologies are tools—they are designed by people and for people. While personalized health technologies are pregnant with potential, the extent to which they affect the health of all populations remains limited. With the current user base, any health benefits derived from using personalized health technologies or future iterations will disproportionately accrue to the affluent unless strategies are adopted to widen access among disadvantaged groups. In time, new business models are likely to emerge that reduce costs, increase affordability, and expand access. Until then, the combination of frugal innovation with public and private sector action can leverage personalized health technologies to advance global health responsibly, sustainably, and equitably.

## Abbreviations

UNICEF: United Nations Children’s Fund
